# Fabrication of easy separable and reusable MIL-125(Ti)/MIL-53(Fe) binary MOF/CNT/Alginate composite microbeads for tetracycline removal from water bodies

**DOI:** 10.1038/s41598-021-03428-z

**Published:** 2021-12-10

**Authors:** Ahmed M. Omer, Eman M. Abd El-Monaem, Gehan M. El-Subruiti, Mona M. Abd El-Latif, Abdelazeem S. Eltaweil

**Affiliations:** 1 Polymer Materials Research Department, Advanced Technology and New Materials Research Institute (ATNMRI), City of Scientific Research and Technological Applications (SRTA-City), P. O. Box: 21934, New Borg El-Arab City, Alexandria, Egypt; 2 Chemistry Department, Faculty of Science, Alexandria University, Alexandria, Egypt; 3 Fabrication Technology Department, Advanced Technology and New Materials Research Institute (ATNMRI), City of Scientific Research and Technological Applications (SRTA-City), P. O. Box: 21934, New Borg El-Arab City, Alexandria, Egypt

**Keywords:** Chemistry, Materials science, Nanoscience and technology

## Abstract

In this investigation, we aimed to fabricate easy separable composite microbeads for efficient adsorption of tetracycline (TC) drug. MIL-125(Ti)/MIL-53(Fe) binary metal organic framework (MOF) was synthetized and incorporated with carbon nanotube (CNT) into alginate (Alg) microbeads to form MIL-125(Ti)/MIL-53(Fe)/CNT@Alg composite microbeads. Various tools including FTIR, XRD, SEM, BET, Zeta potential and XPS were applied to characterize the composite microbeads. It was found that the specific surface area of MIL-125(Ti)/MIL-53(Fe)/CNT@Alg microbeads was 273.77 m^2^/g. The results revealed that the adsorption of TC augmented with rising CNT proportion up to 15 wt% in the microbeads matrix. In addition, the adsorption process followed the pseudo-second-order and well-fitted to Freundlich and Langmuir models with a maximum adsorption capacity of 294.12 mg/g at 25 ◦C and pH 6. Furthermore, thermodynamic study clarified that the TC adsorption process was endothermic, random and spontaneous. Besides, reusability test signified that MIL-125(Ti)/MIL-53(Fe)/CNT@Alg composite microbeads retained superb adsorption properties for six consecutive cycles, emphasizing its potentiality for removing of pharmaceutical residues.

## Introduction

Presently, the scarcity of drinking water is the major problem that is sweeping the world, menacing humanity with annihilation^1,2^. During the turbulent period of COVID-19, the medical staff is exerting great efforts to preserve humanity. However, the tons of pharmaceutical residues especially antibiotics that is being disposing daily into water bodies may be the seed to an even more ferocious pandemic. Thence, it is inevitable to find out effective strategies for removing these noxious pharmaceutical residues from water^3^. In this regard, antibiotics such as tetracyclines (TCs) have been recommended in new research that they may be able to treat COVID-19 infection through their anti-inflammatory and anti-apoptotic activities^4–6^. However, humans could not completely metabolize TCs and around 50–80% of the applied dosage is secreted via urine^7^. Thence, numerous developing techniques have been used for TCs removal from wastewater including; adsorption^8,9^, ultrasonic irradiation^10^, photocatalytic degradation^11–13^, membrane process^14^, and fenton oxidation^15^. Among the mentioned techniques; adsorption has been considered as the most favorable technique for the removal of TCs from wastewater owing to it is simple, economic, low-energy consumption, etc.^16,17^.

Metal organic frameworks (MOFs) is a new brilliant class of crystalline materials that has increasingly drawn a vast consideration owing to its versatile applications^18,19^. Notably, owing to the unique characteristics of MOFs such as water stability, ultrahigh porosity, easy functionalization, thermal stability and high surface area, MOFs have successfully been utilized for the adsorptive removal of heavy metals^20^, pharmaceutical contaminants^21^ and synthetic dyes^22^. One of iron-based MOFs that has been exhibiting notable adsorptive behavior is MIL-53 owing to its structure flexibility, stability in water and chemical stability^23^. Furthermore, MIL-125 is one more bright member in MIL-family that possesses promising photo-catalytic and adsorptive behavior due to its photo-reactivity, thermal and chemical stability, etc.^24^. Although, the individual features of MOFs, there is a huge obstacle to apply them in practical applications which is the difficulty of their recycling as well as their difficult separation from the adsorption mediums. Fabrication of the shaping MOFs like membrane, fiber and beads is considered an effective solution to get rid of MOFs drawback^25,26^. Sodium alginate (Alg); is a water-soluble anionic polysaccharide that is smoothly extracted from brown seaweed^27^. Alginate has acquired huge fame owing to its unique merits such as biodegradability, nontoxicity, strong gelation, biocompatibility, high chemical stability, chelating ability and possession of abundant function groups onto its surface (i.e. hydroxyl and carboxyl)^28–31^. Therefore, Alg has been considered a premium-supporting host of chemical and biological compounds in several potential applications including pharmaceutical, biomedical and especially in wastewater treatment owing to its ability to capture the cationic ions from the target contaminants whether heavy metals or dyes via ion-exchange mechanism^27,32,33^.

The remarkable features of carbon nanotubes (CNTs) including high mechanical strength, high surface area, low cost and its ability to form strong bonds with other molecules or atoms, make them promising candidates for the adsorptive removal of diverse pollutants from wastewater^34–37^. Moreover, multi-walled CNTs are higher accessible and lower cost than single-walled CNTs which render multi-walled CNTs more favorable for the potential applications than single-walled CNTs^38^.

Herein, we aimed to fabricate a novel binary MOF/ CNT composite embedded into Alg microbeads to facilitate the separation of MIL-125(Ti)/MIL-53(Fe) binary MOF after the adsorption process as well as ameliorate its efficiency and reusability. The fabricated MIL-125(Ti)/MIL-53(Fe)/CNT@Alg composite microbeads were characterized by bountiful tools as well as their adsorption aptitude towards TC was assessed utilizing a batch adsorption technique. The kinetics, isotherms and thermodynamics were adequately studied. Furthermore, to prove the economic viability of the fabricated MIL-125(Ti)/MIL-53(Fe)/CNT@Alg composite microbeads, recyclability test was investigated.

## Experimental part

### Materials

Ferric chloride hexahydrate (FeCl_3_⋅6H_2_O) and carbon nanotubes (multi-walled type) were purchased from Alpha Chemika (India). Titanium isoproproxide (TBOT), sodium alginate (NaC_6_H_7_O_6_; medium viscosity) and N,N dimethyl formamide (DMF) were obtained from Shanghai Chemical Reagent (China). Tetracycline and 1,4-benzene dicarboxylic acid (BDC) were bought from Loba Chemie Ltd (India). Ethanol, ammonium solution (NH_4_OH) and dimethyl sulfoxide (DMSO) were provided by Ninghai Jiahe (China).

### Synthesis of MIL-125(Ti)

MIL-125(Ti) was fabricated using a modified procedure reported by Yang et al.^39^, Typically, 1.990 g BDC was dissolved into 50 mL DMF and then 2.7 mL TBOT was slowly added. The reaction solution was transferred into a 100 mL autoclave and heated at 140 °C for 20 h. The resultant white solid was separated by centrifugation, washed with DMF and methanol and dried in oven at 80 °C for 24 h.

### Synthesis of MIL-53(Fe)

MIL-53(Fe) was fabricated according to the previous reported procedure by Yu et al., with slight modifications^23^. Exactly, 0.679 g FeCl_3_.6H_2_O and 0.415 g BDC were dissolved in 50 mL DMF and then kept under mechanical stirring for 15 min. The reaction mixture was transferred into a 100 mL autoclave and heated at 140 °C for 20 h. Finally, the yellow product was separated by centrifugation, washed with DMF and methanol and dried in oven at 80 °C for 24 h.

### Synthesis of MIL-125(Ti)/MIL-53(Fe) binary MOF

MIL-125(Ti)/MIL-53(Fe) binary MOF was fabricated as follows; exactly, 0.679 g FeCl_3_.6H_2_O and 0.415 g BDC were dissolved in 50 mL DMF and then the reaction mixture was kept under continuous stirring at 60 °C for 2 h. In another container, 1.990 g BDC was dissolved in 50 mL DMF and then 2.7 mL TBOT was added drop by drop under vigorous stirring followed by stirring at 60 °C for 2 h. Next, the two solutions were mixed, then transferred to 500 mL autoclave and heated at 140 °C for 20 h. finally, the obtained powder was collected by centrifugation, washed and dried at 100 °C for 24 h.

### Fabrication of MIL-125(Ti)/MIL-53(Fe)/CNT@Alg composite microbeads

MIL-125(Ti)/MIL-53(Fe)/CNT@Alg composite beads were fabricated as follows; Alg was dissolved into 50 mL (5% wt/v) dist. H_2_O under robust mechanical stirring until a clear jelly solution was formed. In another beaker, 5 g MIL-125(Ti)/MIL-53(Fe) binary MOF and CNT (5, 10, 15 and 20 wt%) were soaked into 50 mL dist.H_2_O and sonicated for 2 h. Then after, the homogenous solution of MIL-125(Ti)/MIL-53(Fe) binary MOF/CNT was added to Alg solution dropwise and stirred for 1 h. MIL-125(Ti)/MIL-53(Fe) binary MOF/CNT/Alg solution was added by syringe into a pre-prepared gelling agent of CaCl_2_ (2% wt/v) and kept the obtained microbeads under slow stirring for 1 h. Finally, MIL-125(Ti)/MIL-53(Fe)/CNT@Alg composite microbeads were collected, washed several times with distilled water and dried at room temperature.

**Figure 1:**
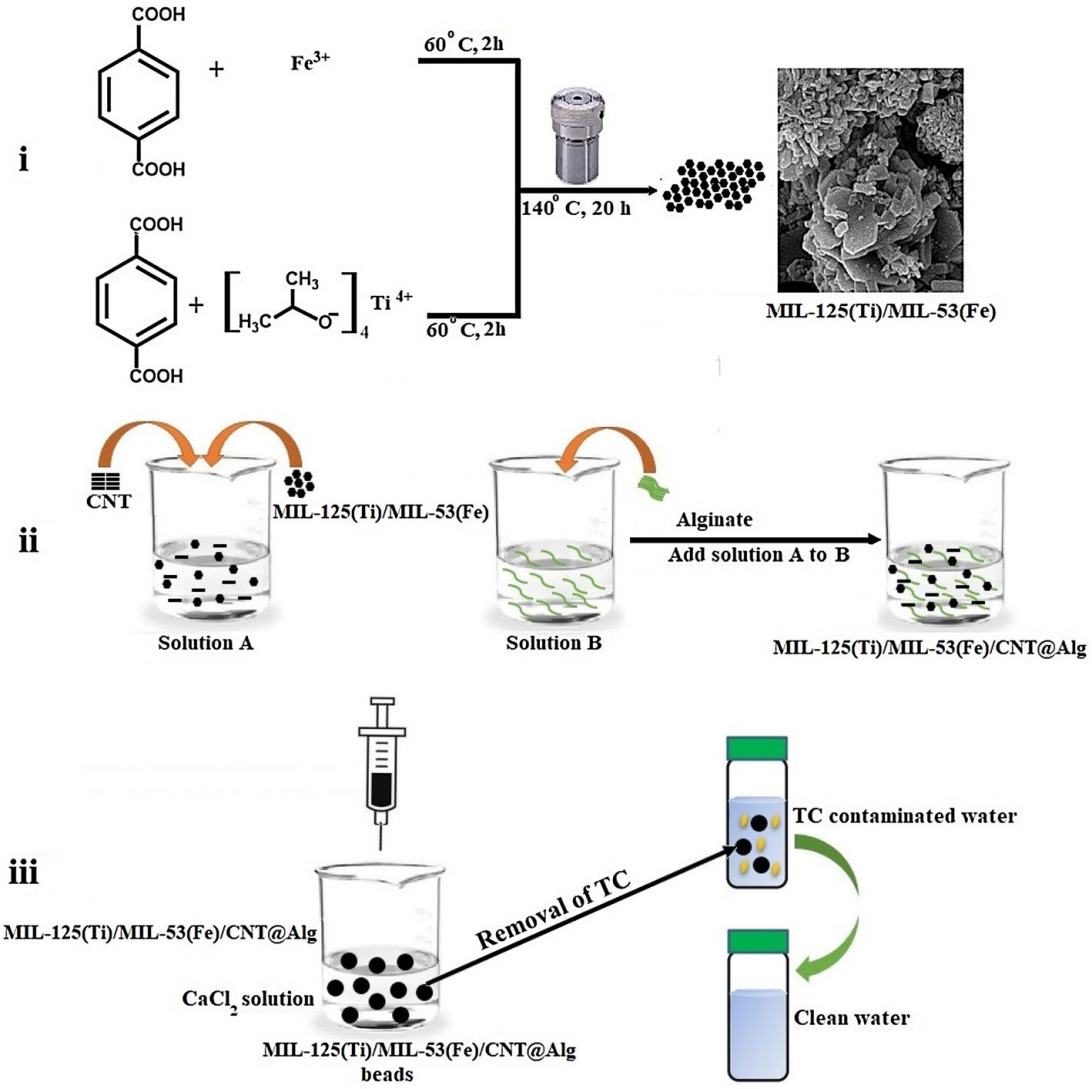
Schematic representation for the fabrication of MIL-125(Ti)/MIL-53(Fe)/CNT@Alg composite microbeads and their adsorption process for TC removal.

Figure 1 represents a schematic representation for the fabrication of MIL-125(Ti)/MIL-53(Fe)/CNT@Alg composite beads.

### Characterization

MIL-125(Ti)/MIL-53(Fe)/CNT@Alg composite microbeads were thoroughly characterized by; Fourier Transform Infrared spectra (FTIR- Tensor II, Bruker) to investigate the chemical composition of the synthesized microbeads. Furthermore, X-ray diffractometer (XRD- MAC Science M03XHF) was used to distinguish the crystal phase. The surface morphology was identified by a Scanning Electron Microscope (SEM- Hitachi-S4800). Besides, X-ray photoelectron spectroscopy (XPS- Thermo scientific-ESCALAB-250Xi VG) was employed to clarify the elemental compositions of the adsorbent. The specific surface area of composite microbeads was measured by Bruner-Emmett-Teller method (BET- Beckman coulter SA3100), while their surface charges were determined by Zeta potential (ZP- Malvern-UK).

### Batch experiment

The key parameters that affect the efficiency of the TC adsorption onto MIL-125(Ti)/MIL-53(Fe)/CNT@Alg composite microbeads were studied precisely using batch mode. During the whole adsorption experiments TC solution was wrapped with aluminum foil to prevent photodegradation of TC. For specifying the optimum pH, 20 mg of dry adsorbent microbeads were soaked into 25 mL TC solution at pH range from 2 to 10 and stirred for 60 min under agitation rate 150 rpm. While, for investigating the effect of adsorbent dose, various doses of MIL-125(Ti)/MIL-53(Fe)/CNT@Alg composite microbeads at range from 10 to 80 mg were added to TC solution at the identified optimum pH. Furthermore, the TC adsorption isotherm was studied at an initial concentration range from 50 to 300 mg/L. Besides, the thermodynamic study was executed at a temperature range from 25 to 55 °C. At a set time, the un-adsorbed TC concentration was evaluated by withdrawing a sample and measured using spectrophotometry at 354 nm. The removal (%) and adsorption capacity (q) were computed by the following equations;

1 $$\mathrm{R \%}=\frac{{\mathrm{C}}_{0 }-{\mathrm{C}}_{\mathrm{t}}}{{\mathrm{C}}_{0}} \times 100$$

2 $${\mathrm{q}}_{\mathrm{e}}=\frac{{(\mathrm{C}}_{0}-{\mathrm{C}}_{\mathrm{t}})\times \mathrm{V}}{\mathrm{w}}$$

where C_0_ and C_t_ symbolize the TC initial concentration and its concentration at certain time, respectively. While, V and w symbolize the TC solution volume and the weight of MIL-125(Ti)/MIL-53(Fe)/CNT@Alg composite microbeads, respectively.

### Reusability study

Undoubtedly, regeneration behavior is one of the main criteria for choosing an adsorbent. Consequently, the reusability study was implemented for five successive cycles as follows; after each adsorption process, MIL-125(Ti)/MIL-53(Fe)/CNT@Alg composite microbeads were collected and soaked in (50 mL, 0.01 M) NaOH under constant stirring. After 1 h, MIL-125(Ti)/MIL-53(Fe)/CNT@Alg composite microbeads were collected and examined in the next cycle.

## Results and discussion

### Characterization of MIL-125(Ti)/MIL-53(Fe)/CNT@Alg composite beads

#### XRD

**Figure 2:**
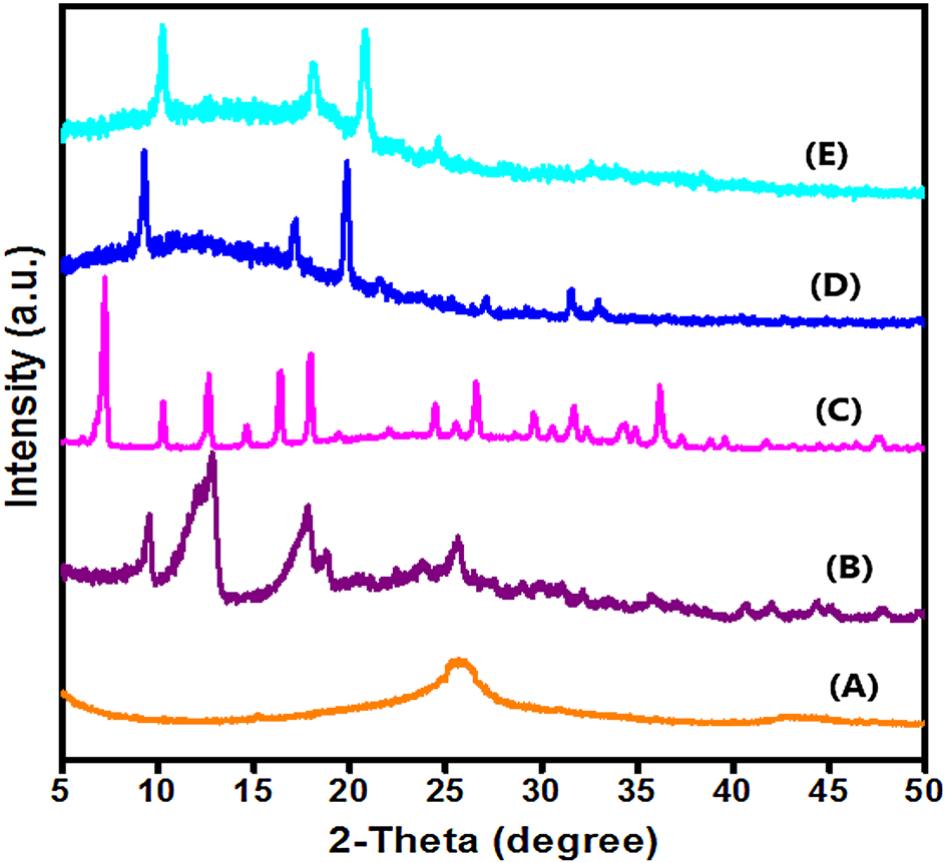
XRD patterns of (**A**) CNT, (**B**) MIL-53(Fe), (**C**) MIL-125(Ti), (**D**) MIL-125(Ti)/MIL-53(Fe) binary MOF and (**E**) MIL-125(Ti)/MIL-53(Fe)/CNT@Alg composite microbeads.

Figure 2 depicts XRD patterns of CNT, MIL-53(Fe), MIL-125(Ti), MIL-125(Ti)/MIL-53(Fe) binary MOF and MIL-125(Ti)/MIL-53(Fe)/CNT@Alg composite microbeads. Moreover, XRD pattern of CNT (Fig. 2A) illustrates the distinguishing peaks of CNT at 2θ = 25.81° and 43.28 which correspond to 100 and 101 planes, respectively^40^. The XRD patterns show the crystalline structure of pure MIL-53(Fe) (Fig. 2B) and MIL-125(Ti) (Fig. 2C) which have been studied extensively in previous literature^39,41^. Moreover, Fig. 2D implies the successful fabrication of MIL-125(Ti)/MIL-53(Fe) binary MOF at which the peak at 2θ = 9.4° may be attributed to overlapping the distinguishing peaks of MIL-125 and MIL-53. Furthermore, MIL-125(Ti)/MIL-53(Fe) binary MOF reveals almost similar degree of crystallinity, agreeing with the studu by Azhar et al.^42^. Besides, XRD pattern of MIL-125(Ti)/MIL-53(Fe)/CNT@Alg (Fig. 2E) infers the successful combination of MIL-125(Ti)/MIL-53(Fe), CNT and Alg since the distinctive peak of CNT emerged with a slight decrease in the intensity of the peaks of MIL-125(Ti)/MIL-53(Fe), confirming that the binary was not destroyed by CNT. This finding agrees with the study by Xiong et al.^43^. In addition, there is no characteristic peak to Alg owing to its amorphous phase^28^. This result is consistent with the study by Eltaweil et al.^2^.

#### FTIR

**Figure 3:**
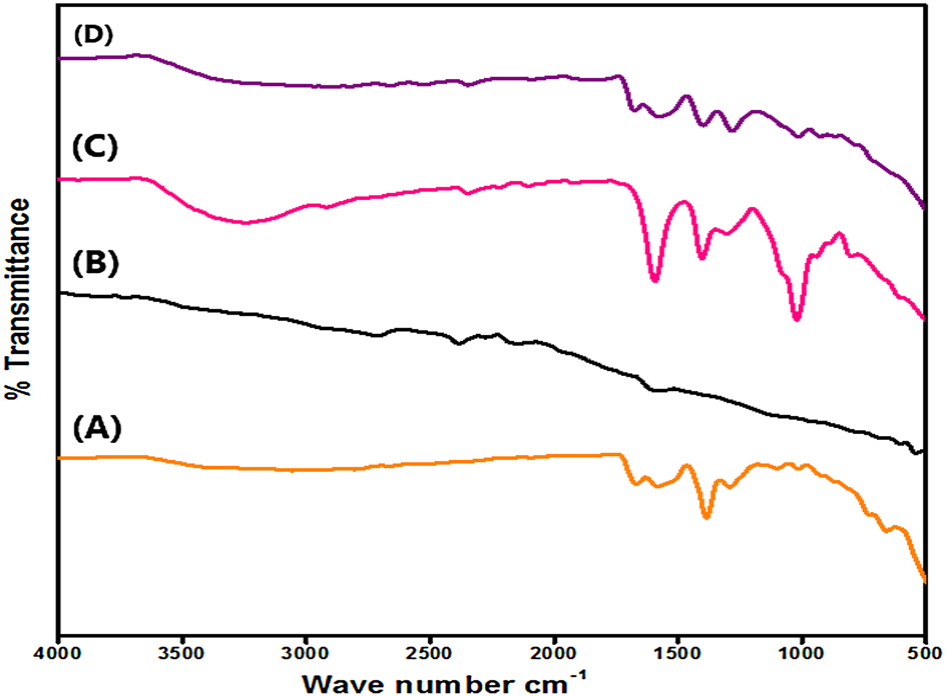
FTIR spectra of (**A**) MIL-125(Ti)/MIL-53(Fe) binary MOF, (**B**) CNT, (**C**) alginate and (**D**) MIL-125(Ti)/MIL-53(Fe)/CNT@Alg composite microbeads.

In order to thoroughly investigate the functional groups of the fabricated MIL-125(Ti)/MIL-53(Fe)/CNT@Alg composite microbeads and the pristine components, FTIR analysis was executed and the resulted are presented in Fig. 3. FTIR spectrum of MIL-125(Ti)/MIL-53(Fe) binary MOF (Fig. 3A) depicts the main distinctive peaks of both MIL-125 and MIL-53. The bands at around 650 and 735 cm^−1^ could be attributed to Fe–O and Ti–O bending vibrations^44,45^. Whereas, the bands at 1101 and 1291 cm^−1^ are assigned to C-H and C-O, respectively^46^. Besides, the two bands at 1385 and 1581 cm^−1^ are ascribed to the vibration of the carboxyl group of BDC that coordinates to the metal centers (i.e. Ti and Fe)^39^. Figure 3B depicts the distinguishing bands of CNT at 1650, 2330 and 2675 cm^−1^ which are attributed to C = C, the formed H-bond and C-H^47,48^. In the Alg spectrum (Fig. 3C), the band at 799 cm^−1^ is related to C-H vibration of pyranose, while the band at around 2916 cm^−1^ is ascribed to C-H stretching vibration. Besides, the band at 1019 cm^−1^ is ascribed to C-O stretching and the band around 3250 cm^−1^ belongs to OH stretching vibration^49,50^. In addition, the belonging peaks to asymmetric and symmetric COO^–^ group emerged at 1401 and 1592 cm^−1^, respectively. Besides, the observed band at 2330 cm^−1^ is assigned to CO_2_ group^51^. Figure 3D clarifies the main characteristic bands of the pristine components, suggesting the successful combination between them.

#### SEM

**Figure 4:**
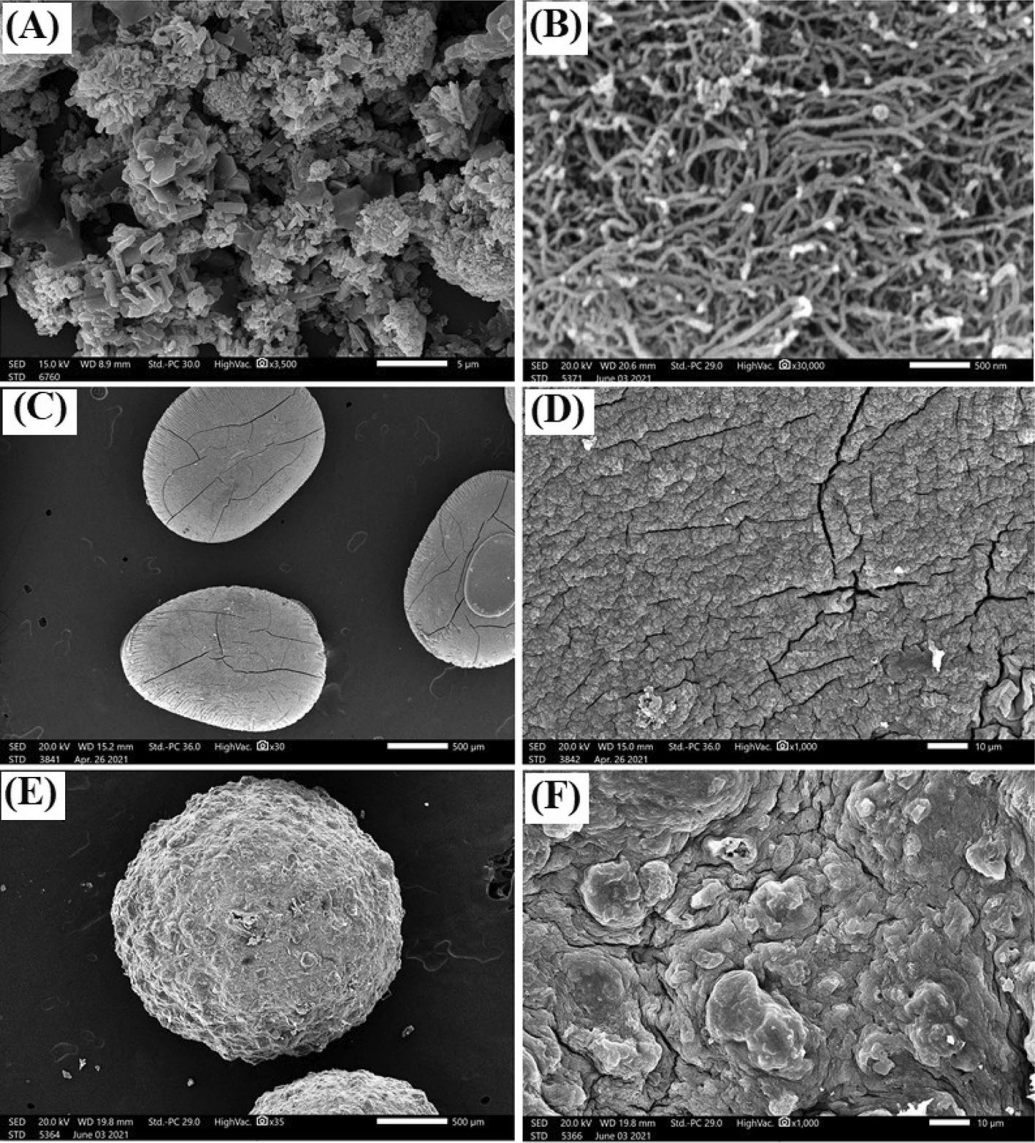
SEM images of (**A**) MIL-125(Ti)/MIL-53(Fe) binary MOF, (**B**) CNT (**C**, **D**) alginate microbeads and (**E**, **F**) MIL-125(Ti)/MIL-53(Fe)/CNT@Alg composite microbeads.

Figure 4A points out aggregated quasi-spherical particles of MIL-125(Ti)/MIL-53(Fe)/CNT@Alg. Whereas, Fig. 4B depicts the typical cylindrical shape of CNT in nano size. Furthermore, SEM image of pristine Alg microbeads (Fig. 4C) reveals that the beads have an elongated shape. In addition, the closer image of the surface of Alg microbeads (Fig. 4D) clarifies a rugged surface with large crevices, reflecting the low mechanical strength that causes the collapse of Alg layers during dehydration. On the other hand, (Fig. 4E) shows a perfectly spherical shape of the fabricated MIL-125(Ti)/MIL-53(Fe)/CNT@Alg composite microbeads. Furthermore, the surface of the microbeads (Fig. 4F) has no fissures, assorting an ameliorated mechanical strength of MIL-125(Ti)/MIL-53(Fe)/CNT@Alg composite microbeads compared to pristine Alg microbeads.

#### XPS

**Figure 5:**
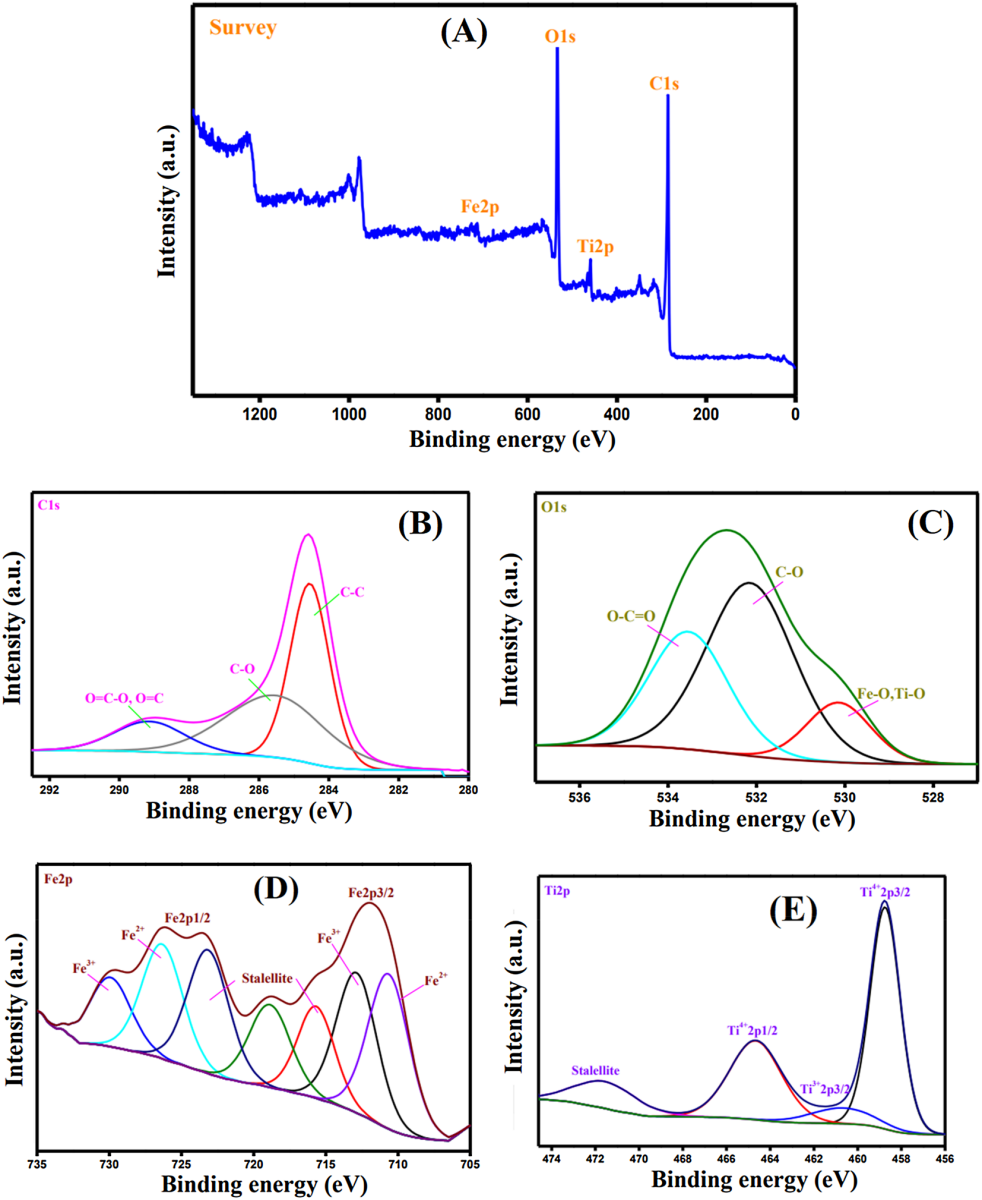
XPS spectra (**A**) wide scan, (**B**) C1s, (**C**) O1s, (**D**) Fe2p, and (**E**) Ti2p of MIL-125(Ti)/MIL-53(Fe)/CNT@Alg composite microbeads.

XPS was utilized for an in-depth inspection of the elemental composition of MIL-125(Ti)/MIL-53(Fe)/CNT@Alg composite microbeads and assuring from the successful combination of the fabricated matrix. It is apparent from the XPS survey (Fig. 5A) that MIL-125(Ti)/MIL-53(Fe)/CNT@Alg consists of four main elements; C1s, O1s, Fe2p and Ti2p. The C1s-spectrum (Fig. 5B) points out peaks at 284.54, 285.54 and 289.14 eV which are ascribed to the carbon-containing groups in MIL-125(Ti)/MIL-53(Fe)/CNT@Alg C–C, C-O and O = C-O/O = C, respectively^52,53^. Moreover, the O1s-spectrum (Fig. 5C) obviously clarifies the belonging peak to M–O (i.e. M; Ti or Fe) at BE of 532.14 eV, proving the formation of both MIL-125 and MIL-53. Besides, the appearance of peaks at 530.14 and 533.54 eV are related to C-O and O = C-O, respectively^54,55^. In addition, Fe2p-spectrum (Fig. 5D) infers the existence of Fe^2+^ and Fe^3+^ at which the related peaks to Fe^2+^ revealed at 710.66 and 756.33 eV. While pertinent peaks to Fe^3+^ at 712.88 and 729.33 eV^56^. Besides, the Ti2p-spectrum (Fig. 5E) shows the relevant peaks of Ti^4+^ (titanium dioxide) at BE of 458.74 and 464.67 eV, as well as the distinctive peaks to Ti^3+^ (titanium suboxide) at BE of 450.68 eV^57^.

#### Textural properties

**Figure 6:**
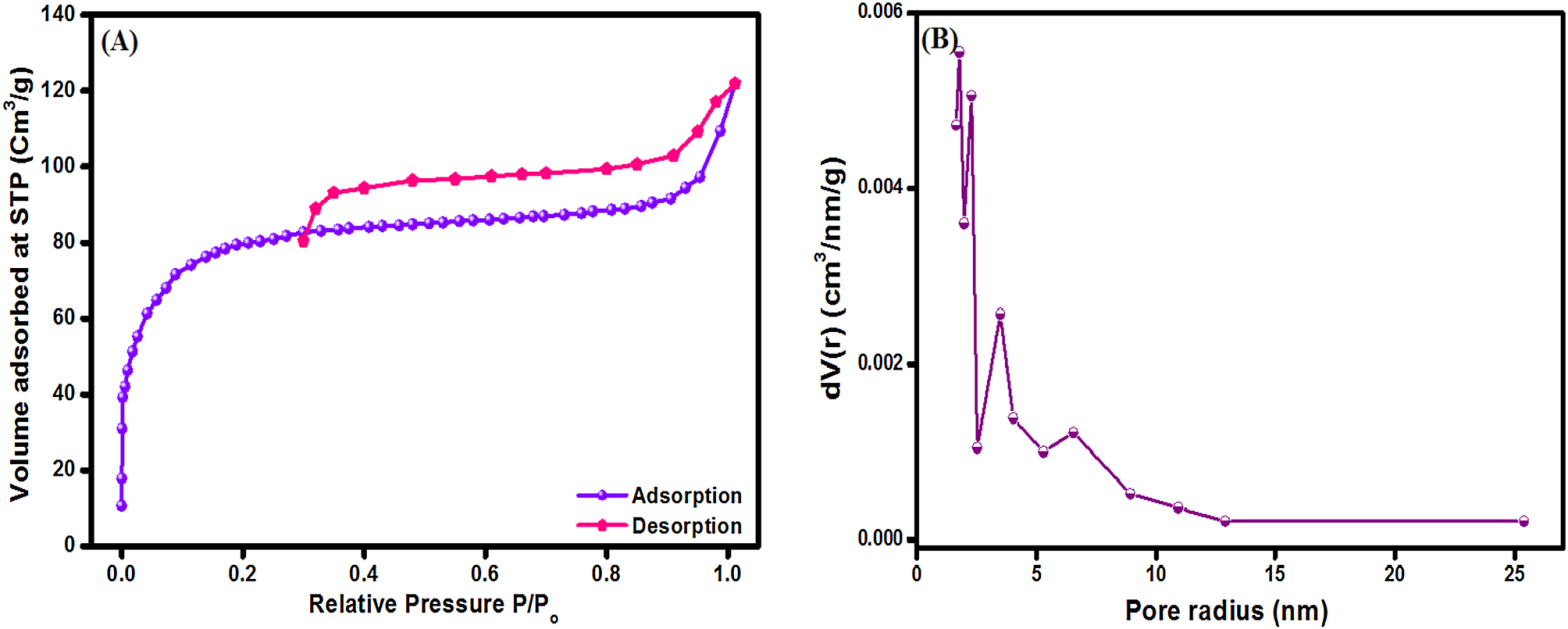
(**A**) N_2_ adsorption/desorption isotherm and (**B**) pore size distribution of MIL-125(Ti)/MIL-53(Fe)/CNT@Alg composite microbeads.

N_2_ adsorption/desorption isotherm of MIL-125(Ti)/MIL-53(Fe)/CNT@Alg is presented in Fig. 6 along with pore size distribution (inset). The MIL-125(Ti)/MIL-53(Fe)/CNT@Alg composite showed type IV isotherm with H_4_ hysteresis loop demonstrating a mesoporous structure of MIL-125(Ti)/MIL-53(Fe)/CNT@Alg microbeads composite. The S_BET_ surface area of MIL-125(Ti)/MIL-53(Fe)/CNT@Alg microbeads was found to be 273.77 m^2^/g with pore volume of 0.0131 cm^3^/g and pore size 2.145 nm.

### Effect of the adsorption conditions

**Figure 7:**
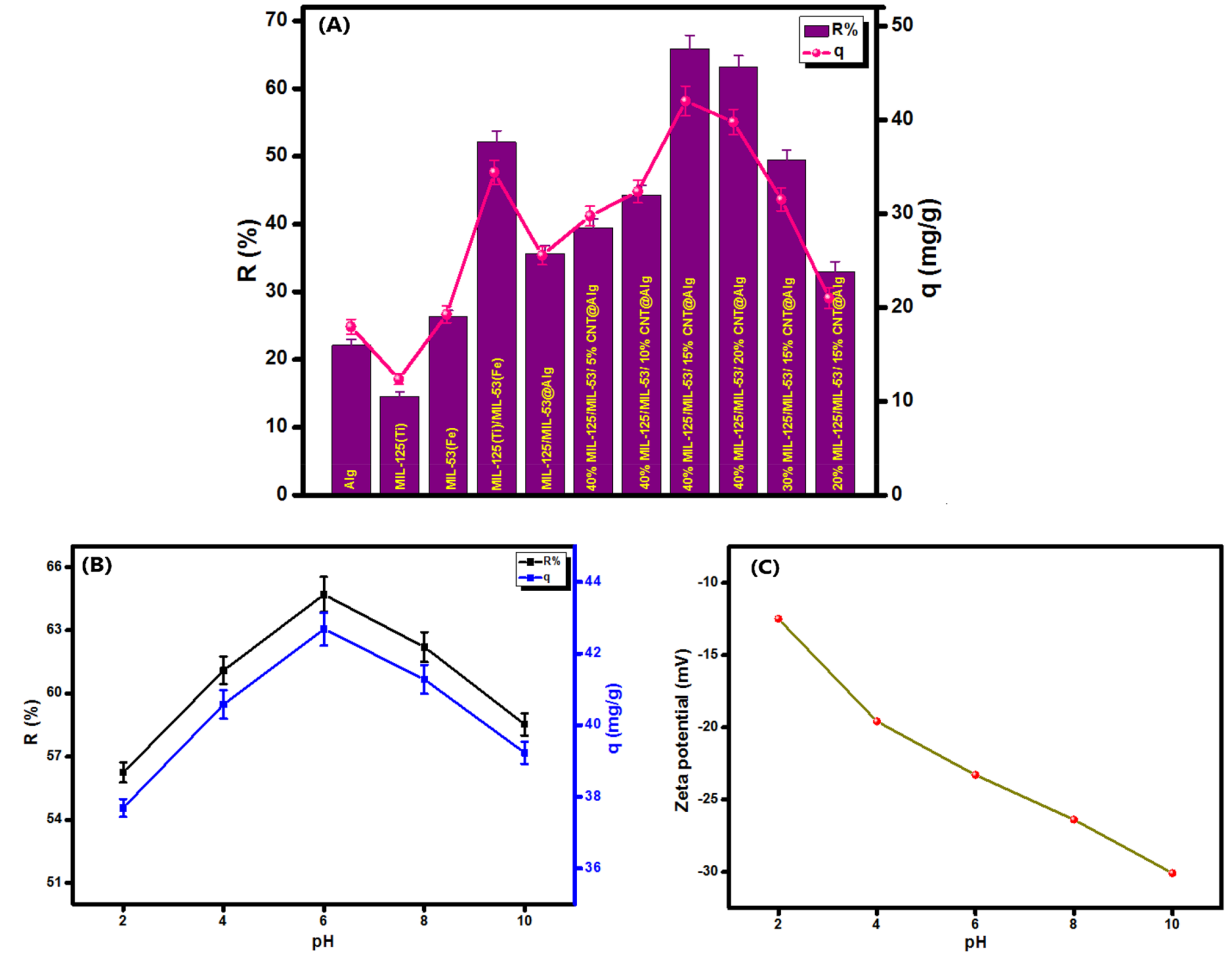
(**A**) Effect of CNT and MIL-125(Ti)/MIL-53(Fe) proportions and (**B**) Effect of pH, Effect of dosage on the removal efficacy and adsorption capacity of MIL-125(Ti)/MIL-53(Fe)/CNT@Alg composite microbeads and (**C**) zeta potential versus pH for MIL-125(Ti)/MIL-53(Fe)/CNT@Alg composite microbeads.

Figure 7A demonstrates the removal efficiency and the adsorption capacity of TC onto MIL-125(Ti)/MIL-53(Fe)/CNT@Alg composite microbeads and their components; MIL-125(Ti), MIL-53(Fe), MIL-125(Ti)/MIL-53(Fe) binary MOF and Alg powder. It was found that the as-fabricated binary MOF exhibits a superb adsorption performance compared with the pristine MOFs. Although, the incorporation of MIL-125(Ti)/MIL-53(Fe) into Alg microbeads solves the separation problem of the binary MOF and boosts its reusability, there was a decline in the adsorption performance of the binary MOF. This finding agrees with the previous study by Zhao et al.^58^. Accordingly, CNT was incorporated into MIL-125(Ti)/MIL-53(Fe)@Alg microbeads to enhance their adsorbability toward TC since CNT act as a platform, boosting the dispersion forces of binary MOF to avoid the particles aggregation^59^. It is apparent that the presence of CNT in the fabricated microbeads has vast leverage since it boosts the adsorptive performance of MIL-125(Ti)/MIL-53(Fe)@Alg composite microbeads toward TC. Furthermore, it was found that the increase in CNT proportion from 5 to 15 wt% increases the removal (%) from 44.20 to 65.10% and the adsorption capacity from 29.76 to 42.02 mg/g. While the excessive CNT proportion over 15 wt% causes a slight decrease in the removal (%) from 65.10 to 61.29% and the adsorption capacity from 42.02 to 39.76 mg/g which may be due to the pore blocking effect, resulting from the excessive CNT proportion in the microbeads matrix^45^. On the other hand, to assert the significance of MIL-125(Ti)/MIL-53(Fe) binary MOF in the amelioration of the adsorption efficacy of MIL-125(Ti)/MIL-53(Fe)/CNT@Alg composite microbeads towards TC, the proportion of MIL-125(Ti)/MIL-53(Fe) was altered from 20 to 40% (Fig. 7A). It was observed that the removal (%) and adsorption capacity values were increased from 32.96% and 21.01 mg/g to 65.10% and 42.02 mg/g with raising the binary MOF proportion from 20 to 40%, respectively. These observations could be a result of improvement the adsorption characteristics of the fabricated MIL-125(Ti)/MIL-53(Fe)/CNT@Alg composite microbeads with more active adsorption sites with increasing the quantity of the synthetized MIL-125(Ti)/MIL-53(Fe) binary MOF in the adsorbent composite matrix^45^. This finding indicates the adsorption aptitude of the novel MIL-125(Ti)/MIL-53(Fe) binary MOF towards TC.

#### Effect of pH

The pH influence on the adsorption behavior of MIL-125(Ti)/MIL-53(Fe)/CNT@Alg composite microbeads towards TC was scrutinized at a pH range from 2 to 10. In fact, TC molecule exists in multi forms, depending on the solution pH at which TC is cationic (TCH_3_^+^) at pH < 3.3, zwitterionic (TCH_2_^0^) at 3.3 < pH < 7.7 and anionic (TCH^−^ or TC^2−^) at pH > 7.7^17^. Figure 7B elucidates an evolution in the adsorption capacity and the removal (%) from 37.69 mg/g and 56.25% to 42.68 mg/g and 64.70%, respectively, with the rising in pH from 2 to 6. Nevertheless, at this pH range, the positive charges on the TC molecule diminish and the negative charges on the surface of the composite microbeads increase from -12.5 to -23.3 mV based on the ZP result (Fig. 7C), inferring that the adsorption of TC onto MIL-125(Ti)/MIL-53(Fe)/CNT@Alg composite microbeads is not dominated by electrostatic interaction. Also, this result is confirmed by the slight diminution in the adsorption capacity and the removal (%) beyond pH 6 from 42.68 mg/g and 64.70% to 39.23 mg/g and 58.63%, respectively. However, the electrostatic repulsion between TC and MIL-125(Ti)/MIL-53(Fe)/CNT@Alg composite microbeads at which TC molecules dwell at this pH region as anionic and the microbeads surface significantly charged with negative charge reach -30.1 mV at pH 10. Accordingly, it was deduced that the adsorption of TC onto MIL-125(Ti)/MIL-53(Fe)/CNT@Alg composite microbeads is not controlled by electrostatic interaction and there is another predominant interaction such as H-bond, hydrophobic interaction, and π-π interaction. These results are consistent with the previous studies by Zhang, Xiong, Gao, and Alatalo^7,43,60,61^.

#### Effect of adsorbent dose

**Figure 8:**
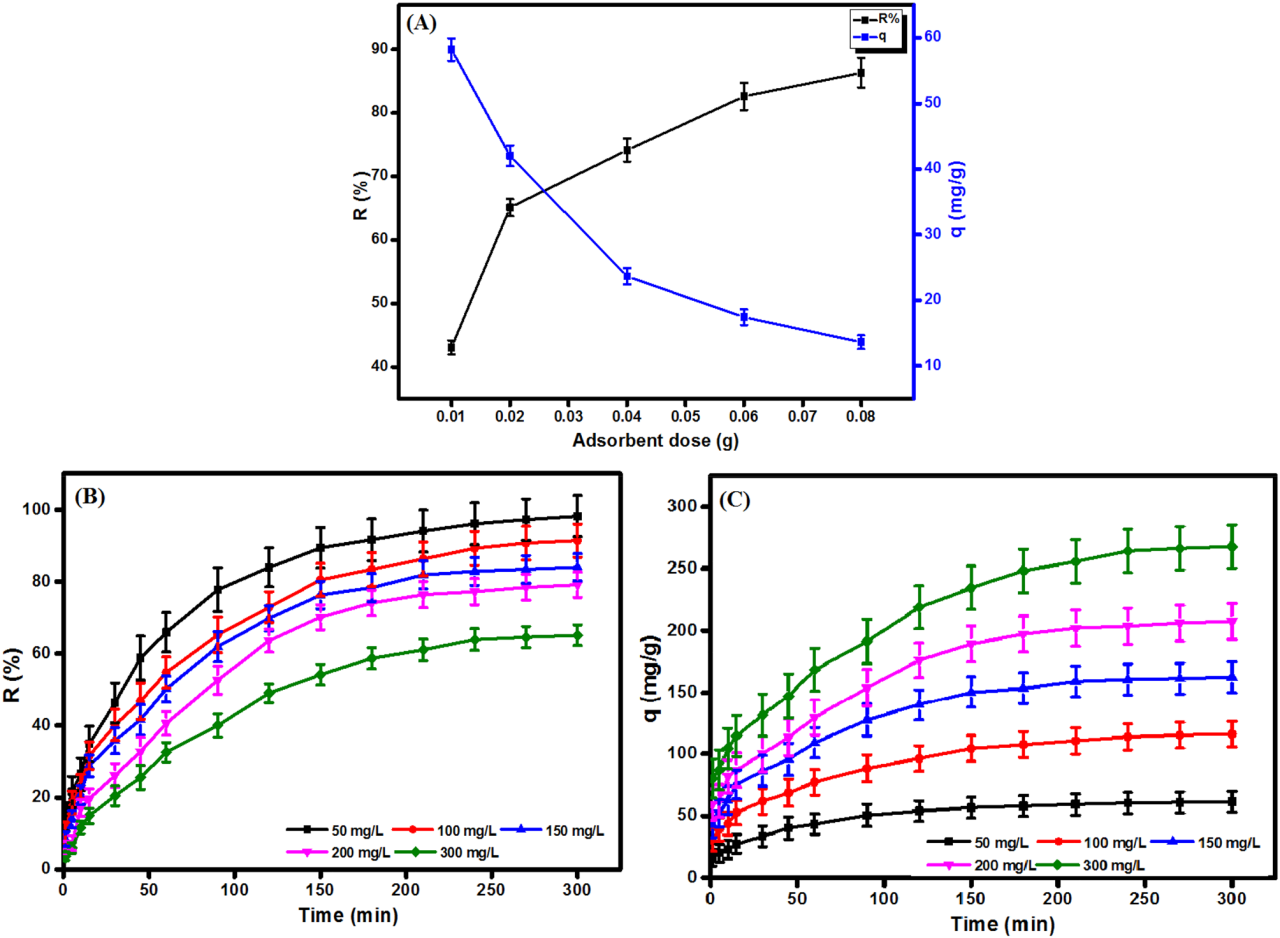
(**A**) Effect of adsorbent dose, (**B**,**C**) effect of initial concentration of the removal efficiency and adsorption capacity of TC onto MIL-125(Ti)/MIL-53(Fe)/CNT@Alg composite microbeads.

Figure 8A points out the impact of the increase in the dose of MIL-125(Ti)/MIL-53(Fe)/CNT@Alg composite microbeads on the removal (%) and adsorption capacity of TC. As expected, the increment in the adsorbent dose from 0.01 to 0.08 g results in an increasing in the removal (%) from 43.08 to 86.33% and a dropping in the adsorption capacity from 58.02 to 13.62 mg/g. This behavior may be interpreted by the increment in the adsorbent dose leads to an increase in the provided active sites that renders the removal (%) goes up. Contrariwise, the adsorption capacity dwindles due to the particles aggregation^25^.

#### Effect of initial TC concentration

Figure 8B demonstrates an increase in the adsorption capacity from 61.52 to 258.10 mg/g with the increase in the TC initial concentration from 50 to 300 mg/L which most likely due to the increase in the driving force of TC molecules towards MIL-125(Ti)/MIL-53(Fe)/CNT@Alg composite microbeads. On the contrary, the increase in the TC initial concentration decreases the removal (%) from 98.19 to 65.08% (Fig. 8C) which may be due to the insufficient active sites on the surface of the microbeads for adsorbing a high concentration of TC.

### Isotherm study

For interpreting the nature of interaction between TC and MIL-125(Ti)/MIL-53(Fe)/CNT@Alg composite micrbeads, the experimental data were comprehensively analyzed by; Langmuir, Freundlich, Temkin and Dubinin-Radushkevich (D-R) isotherm models (Fig. S1). The linear form of These models are expressed as follows^62,63^;

3 $$\mathrm{Langmuir\, equation}: \frac{{\mathrm{C}}_{\mathrm{e}}}{{\mathrm{q}}_{\mathrm{e}}}=\frac{1}{{\mathrm{K}}_{\mathrm{L}} {\mathrm{q}}_{\mathrm{m}}}+\frac{{\mathrm{C}}_{\mathrm{e}}}{{\mathrm{q}}_{\mathrm{m}}}$$

4 $$\mathrm{Freundlich\, equation}:\mathrm{ log}{\mathrm{q}}_{\mathrm{e}}={\mathrm{logK}}_{\mathrm{F}}+\frac{1}{\mathrm{n}}{\mathrm{logC}}_{\mathrm{e}}$$

5 $$\mathrm{Temkin\, equation}: {\mathrm{q}}_{\mathrm{e}}=\mathrm{B lnA}+{\mathrm{BlnC}}_{\mathrm{e}}$$

6 $$\mathrm{D}{-}\mathrm{R equation}:\mathrm{ Ln }{\mathrm{q}}_{\mathrm{e}}=\mathrm{Ln }{\mathrm{q}}_{\mathrm{s}}-{\mathrm{K}}_{\mathrm{ad }}{\upvarepsilon }^{2}$$

where q_e_ and q_m_ the equilibrium adsorption capacity and the monolayer adsorption capacity, respectively. C_e_ is the residual concentraion of TC at equilibrium and K_L_ is Langmuir constant. K_F_ and n are Freundlich constants. $\mathrm{B}=\frac{\mathrm{RT}}{\mathrm{b}}$ , b is Temkin constant related to heat of adsorption and A is the equilibrium binding constant. T is the absolute temperature and R is the gas constant (8.314 J/mol.k). $\upvarepsilon =\mathrm{RT Ln }\left(1+\frac{1}{{\mathrm{C}}_{\mathrm{e}}}\right)$ is the Polanyi potential. K_ad_ is a constant related to mean free energy of adsorption per mole of adsorbate and q_s_ is the saturation adsorption capacity.

According to the R^2^ values (Table S1), the adsorption of TC onto MIL-125(Ti)/MIL-53(Fe)/CNT@Alg composite microbeads fits Freundlich (0.995) and Langmuir (0.993) models, suggesting a monolayer and multilayer adsorption of TC. Furthermore, it was estimated from Langmuir that the maximum adsorption capacity is 294.12 mg/g. Interestingly, n value proves the favorability of the adsorption of TC onto MIL-125(Ti)/MIL-53(Fe)/CNT@Alg composite microbeads where n > 1, as well as the R_L_ values that fall in the range between 0–1, is one more proof to confirm the favorability of the studied adsorption process. Besides, the calculated bonding energy ($E=\frac{1}{\sqrt{2{K}_{ad}}})$ value that less than 8 kJ/mol, confirming that the adsorption of TC onto MIL-125(Ti)/MIL-53(Fe) binary MOF/CNT@Alg composite microbeads is physicosorption^64^.

### Kinetic study

**Figure 9:**
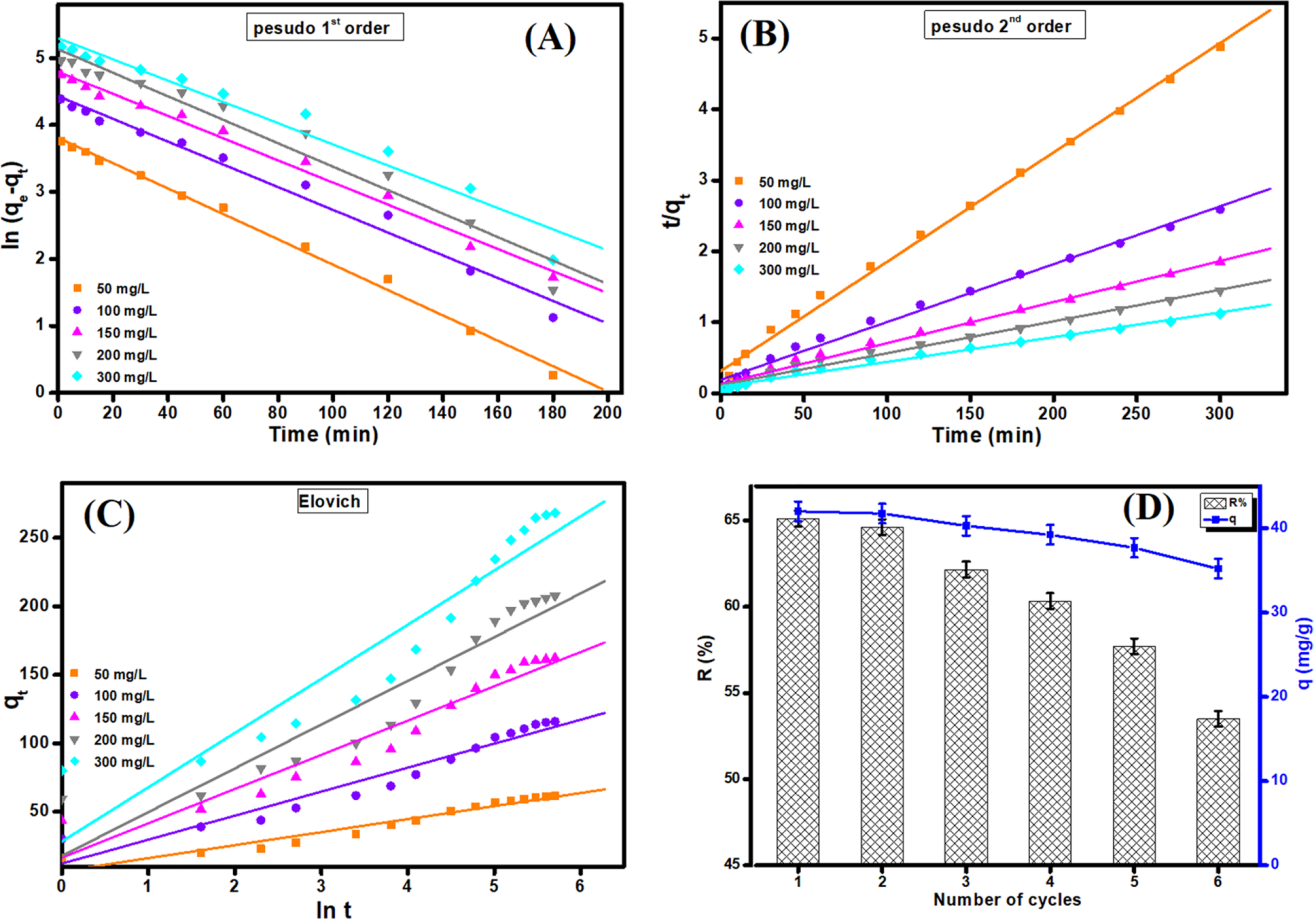
(**A**) Pseudo-first-order, (**B**) Pseudo–second-order and (**C**) Elovich kinetic models for adsorption of TC onto MIL-125(Ti)/MIL-53(Fe)/CNT@Alg composite microbeads. (**D**) Regeneration and reusability of MIL-125(Ti)/MIL-53(Fe)/CNT@Alg composite microbeads.

To deduce the adsorption mechanism of TC onto binary MIL-125(Ti)/MIL-53(Fe) MOF/CNT@Alg composite microbeads, the experimental data were thoroughly modeled by pseudo-first-order, pseudo-second-order and Elovich model (Fig. 9A–C). Equations 7–10 symbolize the linear forms of these kinetic models^62^.

7 $$\mathrm{pseudo-first-order}: {\ln}({\mathrm{q}}_{\mathrm{e}}-{\mathrm{q}}_{\mathrm{t}})={\mathrm{lnq}}_{\mathrm{e}}-{\mathrm{k}}_{1 }\left(\mathrm{t}\right)$$

8 $$\mathrm{pseudo-second-order}: \mathrm{t}/{\mathrm{q}}_{\mathrm{t}}=1/{\mathrm{k}}_{2}{\mathrm{q}}_{\mathrm{e}}^{2}+ 1/{\mathrm{q}}_{\mathrm{e}}\left(\mathrm{t}\right)$$

9 $$\mathrm{Elovich\, model}: {\mathrm{q}}_{\mathrm{t}}=\frac{1}{\upbeta }\mathrm{ ln }\left(\mathrm{\alpha \beta }\right)+ \frac{1}{\upbeta }\ln ({\text{t}})$$

where q_e_ represents the amount of TC that adsorbs onto MIL-125(Ti)/MIL-53(Fe)/CNT@Alg composite microbeads at equilibrium, while q_t_ expresses the amount of TC adsorption at time t. k_1_ is the rate constant of pseudo-first-order and k_2_ is the rate constant pseudo-second- order. α and β are Elovich coefficients that represent the initial adsorption rate and the desorption coefficient, respectively, also relate to the extent of surface coverage and activation energy for chemisorption.

To find out the suitable kinetic model that fits the experimental data there are two main criteria; R^2^ of the suitable kinetic model should be higher than R^2^ of the other applied models as well as the presence of an analogy between q_exp_ and q_cal_ from the suitable model. Accordingly, pseudo-second- order is the most suitable model to represent the adsorption of TC onto MIL-125(Ti)/MIL-53(Fe)/CNT@Alg composite microbeads (Table S2). It is apparent from the computed Elovich coefficients that α values are greater than β values, indicating that the rate of adsorption is higher than desorption^25^.

### Thermodynamic study

As a matter of fact, the change in the process temperature directly affects the nature and the mechanism of adsorption. For deducing the effect of change the temperature from 298–328 K on the adsorption of TC onto MIL-125(Ti)/MIL-53(Fe)/CNT@Alg composite microbeads, Eqs. (10–12) were utilized for calculating the thermodynamics parameter; change in free energy (ΔG°), change in enthalpy (ΔH°) and change in entropy (ΔS°). The negative values of ∆G° (Table 1) prove the spontaneity of this adsorption process.

**Table 1:** Thermodynamic parameters of the adsorption of TC onto MIL-125(Ti)/MIL-53 (Fe) binary MOF/CNT@Alg composite microbeads.

ΔG° (kJ/mol)				ΔH° (kJ/mol)	ΔS° (J/mol K)
298 K	308 K	318 K	328 K	21.11	76.46
−22.77	−23.53	−24.29	−25.06		

10 $${\mathrm{lnK}}_{\mathrm{e}}=\frac{\Delta \mathrm{S}^\circ }{\mathrm{R}}-\frac{\Delta \mathrm{H}^\circ }{\mathrm{RT}}$$

11 $${\mathrm{K}}_{\mathrm{e}}=\frac{{\mathrm{C}}_{\mathrm{Ae}}}{{\mathrm{C}}_{\mathrm{e}}}$$

12 $$\Delta \mathrm{G}^\circ =\Delta \mathrm{H}^\circ -\mathrm{T}\Delta \mathrm{S}^\circ $$

where K_e_ is the thermodynamic equilibrium constant. C_Ae_ is the TC concentration onto the surface of MIL-125(Ti)/MIL-53(Fe)/CNT@Alg composite microbeads, while C_e_ is the concentration of TC in solution at equilibrium. T is the adsorption temperature and R is gas constant.

Figure S2 represents Van't Hoff Plot that elucidates ∆S° and ∆H° from the intercept and slope, respectively. The positive value of ∆S° and ∆H° indicates that the adsorption of TC onto the surface of MIL-125(Ti)/MIL-53(Fe)/CNT@Alg composite microbeads randomness and endothermic, respectively.

### Regeneration study

To assert the viability of our study, the recyclability of the fabricated MIL-125(Ti)/MIL-53(Fe)/CNT@Alg composite microbeads was examined for six consecutive adsorption/desorption cycles. Figure 9D depicts an inconsiderable decrease in the removal (%) and the adsorption capacity from 65.10% and 42.02 mg/g to 53.50% and 35.22 mg/g, respectively, confirming the good recyclability of MIL-125(Ti)/MIL-53(Fe)/CNT@Alg composite microbeads that renders us recommend our novel composite microbeads as a promising candidate for the removal of TC from an aqueous solution.

### Comparison study

To sum, MIL-125(Ti)/MIL-53(Fe)/CNT@Alg composite microbeads possess a superb adsorption capacity toward TC compared with other MOFs-, carbon materials- or alginate beads-based adsorbents (Table 2). This finding suggests that the fabricated composite beads may be utilized in actual wastewater treatment taking into consideration the advantage of their easy separation and remarkable renewability.

**Table 2:** Comparison between the adsorption capacity of MIL-125/MIL-53binary MOF/CNT@Alg composite microbeads and other adsorbent towards the adsorption of TC.

Adsorbent	q_e_ (mg/g)	References
CuCo/MIL-101	54.00	^65^
BM-BC composite	84.54	^66^
UiO-66-(COOH)_2_/GO composite	164.91	^67^
MSABC composite	98.33	^68^
NiCoFe-MOF-74 composite	102.94	^69^
MWCNT/NH_2_-MIL-53(Fe)	368.49	^70^
Alg-Cu beads	53.26	^71^
UiO-66-(OH)_2_/ GO composite	37.96	^72^
MWCNT/MIL-53(Fe) composite	180.68	^43^
Co-SCGBC composite	370.37	^73^
ZIF-8	277.80	^74^
Br-MIL-53(Fe)	309.60	^23^
UiO-66	16.70	^75^
MIL-125(Ti)/MIL-53(Fe) binary MOF/CNT@Alg beads	294.12	This study

### Possible mechanisms for the TC adsorption

Based on ZP measurements and the experimental results of the impact of pH on the TC adsorption aptitude, the adsorption of TC onto MIL-125(Ti)/MIL-53(Fe)/CNT@Alg composite microbeads is not controlled by the electrostatic interaction. Consequently, it is expected that there are other mechanisms that dominate the adsorption process such as; Pore filling effects since the length, width and height of the three-dimensional TC molecules are 1.23, 0.84 and 0.67 nm, respectively, while the average pore size of the microbeads is 2.145 nm. So, the pores of the microbeads are loose enough to penetrate the TC molecules^76^. Besides, π-π interaction between the aromatic rings in MIL-125(Ti)/MIL-53(Fe)/CNT@Alg (π- electron donor) and TC molecules (π- electron acceptor)^7,43^. In addition, Coordination bonds between the unsaturated metals (i.e., Ti and Fe) and TC as well as hydrophobic interaction especially the presence of CNT increases the hydrophobicity of the microbeads^77,78^. Although, many studies suggested H-bonding as one of the controlling mechanisms on the TC adsorption, it is difficult to be the main adsorption interaction. The H-bonding interaction between water molecules and functional groups is much stronger than that between TC and the functional groups of MIL-125(Ti)/MIL-53(Fe)/CNT@Alg composite microbeads^78^.

## Conclusion

All in all, this study presented the fabrication of MIL-125(Ti)/MIL-53(Fe)/CNT@Alg composite microbeads for removing of tetracycline drug residue from aqueous solutions. The formulated adsorbent composite was proved its chemical structure, thermal stability and surface morphology, while batch adsorption experiments were conducted to evaluate its aptitude for adsorption of TC under several optimization conditions. The results clarified that incorporation of CNC into the composite matrix played a significant role in the adsorption process, since the removal (%) of TC was increased with increasing CNC quantity up to 15w%. A sequence of adsorption isotherm models and kinetic studies led us to conclude that the adsorption of TC onto MIL-125(Ti)/MIL-53(Fe)/CNT@Alg composite microbeads process was fitted to Freundlich and Langmuir isotherm model with a maximum adsorption capacity of 294.12 mg/g at 25 ◦C and followed the pseudo-second-order kinetic model, spontaneous. The results of thermodynamic studies clarified that the adsorption process could be described as spontaneous, endothermic and randomness process. Reusability studies confirmed that the developed adsorbent exhibited a superior recycling capability even after sex repeated cycles with good performance for adsorption of TC. Thus, the fabricated adsorbent composite has some operational benefits such as easy separation, decent adsorption performance and better reusability, suggesting its applicability for removing TC residue from aquatic mediums.

## Supplementary Information

Supplementary Information.
