# Supplementary material for: Fabrication of easy separable and reusable MIL-125(Ti)/MIL-53(Fe) binary MOF/CNT/Alginate composite microbeads for tetracycline removal from water bodies
Source: Sci Rep. 2021 Dec 10;11:23818. doi: 10.1038/s41598-021-03428-z (PMC8664953; doi:10.1038/s41598-021-03428-z)
Supplement: Supplementary file 1 — Supplementary Information. [file 41598_2021_3428_MOESM1_ESM.docx]

**Supplementary information**


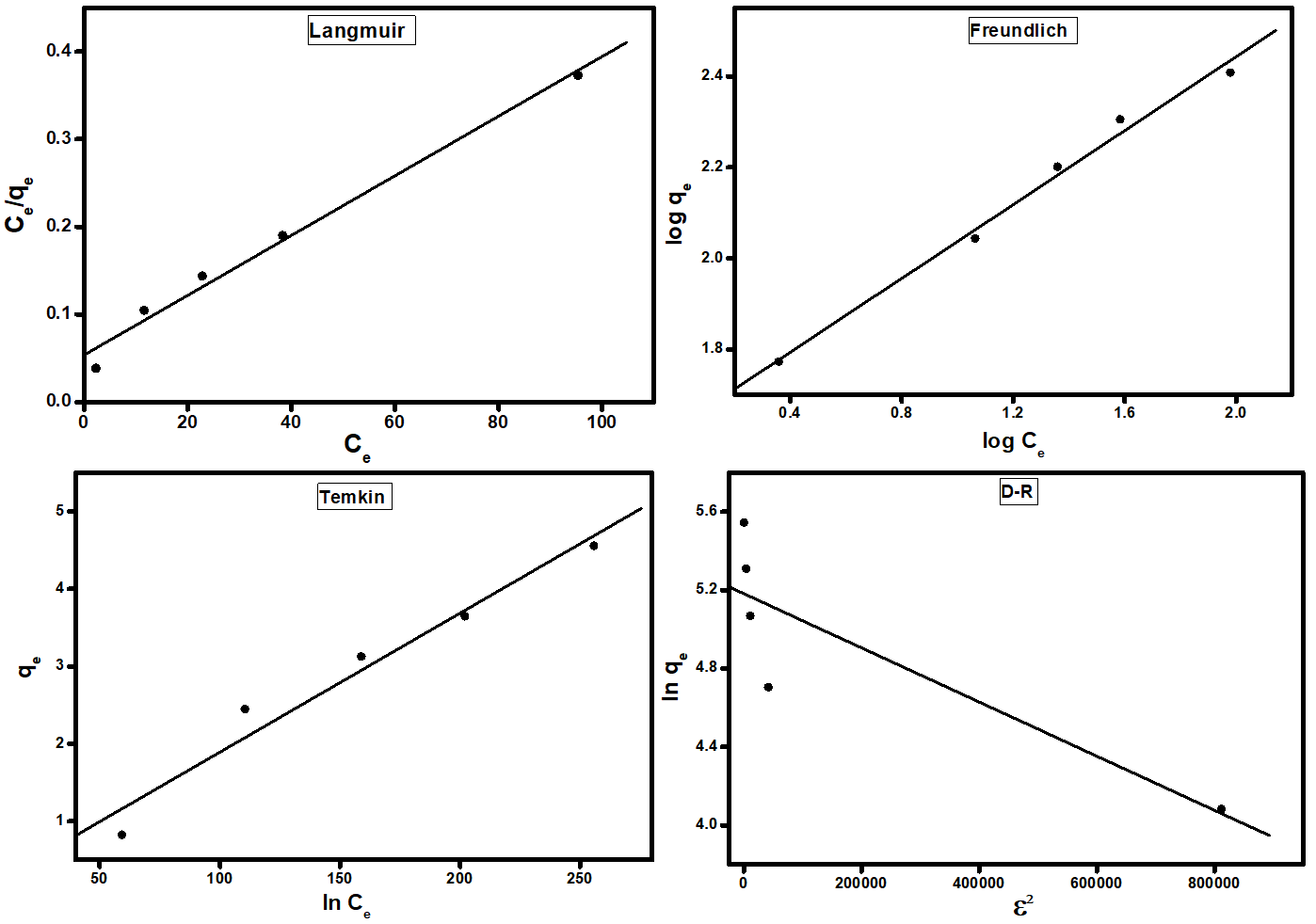


**Figure S1.** Isotherms plots for the TC adsorption onto MIL-125(Ti)/MIL-53(Fe)/CNT@Alg composite microbeads; **(A)** Langmiur, **(B)** Freundlich, **(C)** Temkin and **(E)** D-R.

**Figure S2.** Van’t Hoff diagram for the adsorption of TC onto MIL-125(Ti)/MIL-53(Fe) binary MOF/CNT@Alg composite microbeads.

**Table S1.** The parameters derived from isotherm models for the adsorption of TC onto MIL-125(Ti)/MIL-53(Fe) binary MOF/CNT@Alg composite microbeads

| **Isotherm model** | **Parameter** | **Value** |
| --- | --- | --- |
| **Langmuir** | q_m_ (mg/g) | 294.12 |
|  | b (L/mg) | 0.063 |
|  | R^2^ | 0.993 |
| **Freundlich** | n | 2.265 |
|  | k_F_(L/mg) | 42.806 |
|  | R^2^ | 0.995 |
| **Temkin** | A (L/g) | 1.015 |
|  | B (J/mol) | 53.583 |
|  | b (KJ/mol) | 46.238 |
|  | R^2^ | 0.981 |
| **D-R** | q_s_ | 178.11 |
|  | K_ad_ (mol^2^/K^2^J^2^) | 1x10^-6^ |
|  | R^2^ | 0.863 |

**Table S2.** Adsorption kinetic model parameters of the adsorption of TC onto MIL-125(Ti)/MIL-53(Fe) binary MOF/CNT@Alg composite microbeads

| **Kinetic models and parameters** | **Concentration (mg/L)** | | | | | | | |  |
| --- | --- | --- | --- | --- | --- | --- | --- | --- | --- |
|  | 50 | | | 100 | | 150 | | 200 | 300 |
| q_e, exp_(mg/g) | | 61.52 | 115.95 | | 162.31 | | 207.65 | | 268.10 |
| **Pseudo-first- order** |  | | |  | |  | |  |  |
| q_e,cal_ (mg/g) | 45.07 | | | 48.01 | | 120.83 | | 168.85 | 199.60 |
| k_1_ (min^-1^) | 0.018 | | | 0.017 | | 0.016 | | 0.018 | 0.015 |
| R^2^ | 0.995 | | | 0.981 | | 0.989 | | 0.961 | 0.958 |
| **Pseudo-second-order** |  | | |  | |  | |  |  |
| q_e,cal_ (mg/g) | 64.94 | | | 123.46 | | 172.41 | | 222.22 | 285.71 |
| k_2_ (g.mg^-1^.min^-1^) | 0.007 | | | 0.004 | | 0.003 | | 0.002 | 0.001 |
| R^2^ | 0.996 | | | 0.992 | | 0.993 | | 0.988 | 0.986 |
| **Elovich** |  | | |  | |  | |  |  |
| α (mg/g min) | 20.239 | | | 32.980 | | 43.730 | | 65.319 | 80.940 |
| β (g/mg) | 0.101 | | | 0.059 | | 0.040 | | 0.031 | 0.025 |
| R^2^ | 0.941 | | | 0.935 | | 0.931 | | 0.894 | 0.892 |
